# The Moment of Patient Safety: Iatrogenic Injury, Clinical Error and Cultures of Healthcare in the NHS

**DOI:** 10.1093/shm/hkad089

**Published:** 2024-03-12

**Authors:** Christopher Sirrs

**Affiliations:** Department of History, University of Warwick, Coventry, UK

**Keywords:** patient safety, NHS, iatrogenic injury, clinical error, culture

## Abstract

This article explores the ‘the moment of patient safety’—the period around 2000 when patient safety became a key policy concern of the British National Health Service (NHS), and other healthcare systems. While harm caused by medical care (iatrogenic injury) had long been acknowledged by clinicians and scientists, from 2000 a new systemic language of patient safety emerged in the NHS that promoted novel managerial and regulatory approaches to patient harm. This language reflected the state’s increasing role in regulating healthcare, as well as the erosion of medical autonomy and the rise of new forms of bureaucratic management. Acknowledging a transnational, intellectual context behind the rise of policy interest in patient safety—for example, the application of insights from the industrial safety sciences—this article examines the role played by domestic cultural factors, such as medical negligence litigation and healthcare scandals, in helping to define the new language in Britain.

## Introduction

In 2000, the UK Department of Health (DOH) published *An Organisation with a Memory* (*AOWAM*).^1^ Stemming from an Expert Committee chaired by the Chief Medical Officer, Liam Donaldson, this report painted a worrying picture of the ability of the National Health Service (NHS) to detect and learn from serious healthcare failures. These ranged from the rate of healthcare-associated infections such as MRSA, and suicides in mental health facilities, to incidents involving drugs and medical equipment, and errors in surgery. *AOWAM* responded to a series of high-profile scandals over the preceding decade, which related not only to the quality and safety of care in particular hospitals, but also the competence and conduct of individual clinicians.^2^ They included the deaths of children undergoing heart surgery at Bristol Royal Infirmary; the unethical retention of organs at Alder Hey Children’s Hospital; numerous cases of professional misconduct by doctors (including sexual assault) and the murders committed by the GP, Harold Shipman and nurse, Beverley Allitt.^3^ As the Health Secretary, Alan Milburn noted in his foreword: ‘Too often in the past we have witnessed tragedies which could have been avoided had the lessons of past experience been properly learned’.^4^

Yet, the ‘tragedies’ *AOWAM* addressed were also more insidious and every day. The same mistakes, or errors in clinical care kept being repeated, from the incorrect dosing of drugs, to the misinterpretation of diagnostic instruments and failures in communication leading to patient harm. Systems for recording, analysing and responding to healthcare failures were also deficient. The report estimated that one in ten patients admitted to NHS hospitals, or over 850,000 a year, experienced an ‘adverse health care event’: ‘an event or omission arising during clinical care and causing physical or psychological injury to a patient’.^5^ Up to half of these were deemed to be preventable. Besides the financial cost to the NHS (an estimated £2 billion in additional bed-days alone), such statistics not only called into question the basic function of hospitals—to restore health—but also diminished public trust in the NHS. Behind these statistics were stories of injury and anguish among thousands of patients and families, stories which the British media had reported with alarm over the preceding years. As one victim of a medical ‘blunder’ remarked to *The Observer* in 1993: ‘We regard doctors as gods, but I have lost all faith in the medical profession’.^6^

The publication of *AOWAM* in 2000 marks the key moment when ‘patient safety’ appeared on the policy agenda of the NHS: a commitment to ‘the avoidance, prevention and amelioration of adverse outcomes or injuries stemming from the process of healthcare’.^7^ This article explores the cultural conditions that shaped this moment, asking both why a systemic language of patient safety crystallised in the NHS around 2000, and why, given medicine’s ostensible interest in protecting patients from harm, such a language took so long to appear. The answer, I suggest, lies in understanding various beliefs, attitudes and assumptions prevalent within professional cultures of healthcare in the twentieth century; how these were reflected and embedded in the way health services were managed; and how they began to be questioned as the century ended by various groups, including campaigners, policymakers and reform-minded doctors and managers.

As my first section, ‘Languages of Healthcare Harm’ shows, the problem of harm caused by medical care (iatrogenic injury) had been recognised for centuries. Indeed, healthcare spaces such as hospitals had long been seen as sites of hazard.^8^ As the history of inquests, investigations and inquiries in British healthcare over the previous two centuries shows (from the deaths of patients undergoing anaesthesia in the 1890s, to acts of abuse and neglect in long-stay institutions in the 1960s), poor outcomes, problems or failures in healthcare could attract medical, legal, political and even public scrutiny.^9^ However, as I explore in the following section, ‘The Wall of Silence’, the extent of patient harm, especially attributable to clinical error, was widely hidden from patients and policymakers until recently.^10^ Patients in the NHS faced formidable cultural, legal and organisational barriers that hindered them in obtaining compensation, information, or even an apology following an adverse event. It was not until this ‘culture of silence’ began to erode in the late twentieth century, as medical autonomy declined—and a culture of managerialism expanded—that a comprehensive language of ‘patient safety’ became possible. In particular, the moment of patient safety reflected the state’s expanding role in the regulation of medical care and hospital standards.^11^ This phenomenon is the subject of the final two sections, ‘Clinical Risk Management’ and ‘Regulatory Crisis’.

Patient safety was thus the product of wider changes in the relationship between health professionals, patients and the state. It also reflected changes in how clinicians, managers, policymakers and patients thought about and responded to patient harm. Patient safety, I argue, was not simply an intellectual development, but a cultural one, deeply interwoven with other contemporaneous developments in healthcare. In Britain, many of these developments had their background in the NHS’s structure and organisation since 1948, as well as the shifting power and autonomy of the medical profession. However, from the late 1980s to 2000, several trends came together to shape an embryonic language of patient safety. I highlight deepening public and political scrutiny of medical self-regulation and the accountability of the medical profession; concerns by doctors and politicians about medical negligence litigation; increasing managerial oversight of healthcare; and the impact of scandals in the 1990s (notably the Bristol Heart Scandal) on public perception of safety in the NHS.

As I explain, patient safety is essentially a modern preoccupation. While clinicians and hospital administrators have taken steps over the centuries to protect patients and manage the risks of hospitalisation and treatment, patient safety is novel in terms of the intensity of policy interest and the way it has been embedded in healthcare systems (through systemic regulation, management and monitoring). For this reason, historians have yet to historicise patient safety.^12^ However, this article connects with a wide variety of themes explored by historians. These include (among others) histories of medical harm and risk;^13^ patient complaints;^14^ bioethics^15^ and medical negligence.^16^

Two themes deserve close mention. Firstly, the reconfiguration of the patient as an active consumer in the second half of the twentieth century, as Mold outlines, informed many of the cultural changes discussed in this article.^17^ The decline of public trust in medical authority; the rise of patient advocacy organisations; ideas of consumer protection; and increasing patient litigation all indicate a profound shift in the relationship between patients and health services. This was one where patients increasingly exercised their voice, choice and rights: the word ‘patient’ in ‘patient safety’ suggests patients’ needs or expectations became prioritised in healthcare (as also seen in ‘patient-centred care’), although some have argued patients were marginalised as patient safety became a ‘managerial concept’.^18^

Secondly, scholars have explored complaints as a vehicle by which patients expressed discontentment with care.^19^ However, while responses to complaints provide a useful lens onto attitudes towards patient care in the past, for the reasons discussed in the section ‘The Wall of Silence’, complaints shed little light on actual rates of harm or error in the NHS. First, there is the problem noted by Klein in his classic study *Complaints Against Doctors*: formal complaints were the ‘tip of the iceberg’ of wider discontentment with care.^20^ Patients did not put into writing most grievances against doctors or hospitals, and in any case, Klein’s research suggested communication, rather than actual harm, was their main source of grievance. The sociologist Linda Mulcahy has argued patients faced considerable difficulties in making complaints, and these could be amplified when complaining about complications, or other aspects of treatment.^21^ For example, patients could find complaint procedures confusing and difficult to navigate, and doctors could even pathologise complainants, suggesting they were vindictive or mentally ill. Another issue (discussed below) was that NHS information systems were ineffective at using complaints to improve safety. This highlights how systems and processes were necessary at a national level (such as reporting systems) before lessons at a local, hospital level could be disseminated.

To date, the only major histories of patient safety have been written by figures associated with the ‘patient safety movement’—the coalition of scientists, researchers and campaigners who have studied and promoted safety in healthcare.^22^ This literature is largely US-centric, and has concentrated on intellectual developments, especially in the 1990s, to the emergence of policy interest in patient safety. For example, they have highlighted the importance of scientific studies, such as the Harvard Medical Practice Study, in revealing the extent of harm and error in healthcare systems for the first time.^23^ They have also shown how dramatic language in official reports and journal articles focused minds on the problem.^24^ For example, a study of medical error by the physician Lucian Leape in 1994 suggested the possible level of death resulting from medical harm in the USA was ‘the equivalent of three jumbo-jet crashes every 2 days’.^25^

This growing awareness of patient harm and clinical error resulted in recognition within healthcare policy circles of the need to incorporate lessons from other complex, safety-critical industries into healthcare, namely aviation.^26^ The quote above reveals how aviation became a key comparator to healthcare, with techniques that helped reduce aviation accidents, such as confidential reporting systems, pre-flight checklists and simulation training inspiring approaches to healthcare safety. The desire to learn from safety elsewhere also underpinned efforts to apply insights from the industrial safety sciences: psychology, human factors (ergonomics) and sociology. Such insights included an understanding of the human factors shaping safe and unsafe care, the need for root-cause analysis of critical incidents and the application of system models of accident causation, namely, the British psychologist James Reason’s ‘Swiss cheese’ model.^27^

Britain, therefore, was not alone in experiencing a surge of policy interest in patient safety around 2000. Indeed, patient safety emerged as an international concern.^28^ Existing histories have explored cultural developments in the USA which broadly mirrored developments in Britain, such as the impact of healthcare scandals and concerns about medical malpractice. Further, they have engaged with the sociological literature on professionalism, emphasising how patient safety materialised in the context of an increasingly bureaucratised and managed system of healthcare, where doctors’ autonomy and control over their systems of work declined.^29^ However, these works say little about the emergence of patient safety in Britain (or elsewhere). Acknowledging this transnational context, here, my focus is on the specific cultural and political changes surrounding the NHS, which made a systemic focus on patient safety possible.^30^

## Languages of Healthcare Harm

At face value, the need to explain the emergence of patient safety as an explicit focus of healthcare policy in the NHS may seem strange. After all, patients have undoubtedly experienced harm for as long as medicine has existed. And surely a desire to protect patients is at the heart of clinical practice—what it means to be a good doctor or good nurse? Patient safety may thus seem as old as medicine itself. However, an exploration of the changing language around patient harm in healthcare underlines patient safety as a contemporary historical artefact.

The fact medicine can harm patients, as well as benefit them, has been recognised for millennia.^31^ For example, Sharpe and Faden describe how the Ancient Babylonian Code of Hammurabi (seventh century BCE) referred to errors by physicians, while the Islamic physician Al-Rohawi (ninth century CE) used the ambiguous Greek term *pharmakon* to describe medicine, having connotations of both remedy and poison.^32^ The need to protect patients was reflected in the Hippocratic dictum to ‘do no harm’; however, interpretations of this phrase changed markedly over the centuries, as doctors placed different emphases on the risks and benefits of treatment—the call to *first* do no harm, for example, responded to the drastic and risky interventions justified by proponents of heroic medicine such as Benjamin Rush (1746–1813).^33^

Over the last two centuries, especially, clinicians and scientists have attempted to reduce the risks of treatment and hospitalisation. In the nineteenth century, the hospital itself increasingly became seen as toxic and pathological: memorably, the Scottish surgeon James Simpson (1811–70) remarked patients admitted to hospital were ‘exposed to more chances of death than the English soldier on the field of Waterloo’.^34^ The control of hospital infection from the nineteenth century has been typically taken as the ‘creation myth’ for patient safety, with researchers identifying the work of certain pioneers, such as Semmelweis’ encouragement of handwashing, Nightingale’s promotion of hospital cleanliness and Lister’s development of operative and postoperative antisepsis. The work of the Boston surgeon Ernest Amory Codman (1869–1940) in monitoring surgical outcomes and categorising error, is also invoked as a precursor of concerns about patient safety.^35^ As medicine touched new parts of the body for the first time, medical innovations—whether anaesthesia, X-rays or the contraceptive pill—were also often accompanied by fierce debates about risks and benefits.^36^ However, the response to new medical technologies, or the pioneering work of certain practitioners to reduce medical risk, is different from the broad, high-level way patient safety has been promoted in healthcare since 2000.

By the mid-twentieth century, doctors increasingly recognised the problem of iatrogenic injury. However, it could be tolerated, especially considering rapid developments in medical technology that were improving patient outcomes. In 1956, the American physician Major Robert Moser famously referred to iatrogenic injuries as ‘diseases of medical progress’, while David Barr argued they were the ‘price’ of modern diagnosis and therapy.^37^ In the early 1960s, officials in the British Ministry of Health (MOH) questioned the need for research into hospital accidents such as falls, since they were seen to largely occur among the elderly, and research would not ‘tell us much we do not [already] know’.^38^ Officials in the Department of Health and Social Security later questioned the need to collect statistics on clinical error, arguing not only such data collection would be difficult, but also of ‘doubtful value’.^39^

Generally, the everyday language used by clinicians until recently was not one of patient safety, but harm. Doctors and nurses spoke of ‘medical accidents’, ‘mistakes’, ‘mishaps’, ‘errors’, ‘untoward occurrences’, ‘side-effects’ and ‘complications’. However, the terms ‘patient safety’, ‘safety of patients’ and ‘health and safety of patients’ could be employed in a generic way to reflect the need to protect patients from harm. This applied especially to hazards in the hospital environment, such as fire, falls, trips, slips, burns, infection or radiation exposure. For example, a conference held by the King Edward’s Hospital Fund on ‘hospital safety’ in 1968 used ‘patient safety’ to refer to the protection of patients against environmental hazards, and tellingly, was aimed at hospital administrators rather than clinicians.^40^ Elsewhere, a language of safety, reflecting protection against injury, was used in relation to specific risks, such as the safety of medicines, vaccines and equipment. For instance, following the Thalidomide tragedy, in 1963, the MOH established the Committee on the Safety of Drugs (later Medicines), which resulted in the development of the Yellow Card system for reporting adverse drug reactions.^41^

Linguistically, however, the term ‘patient safety’ was popularised only from the mid-1990s.^42^ In Britain, the emergence of an explicit language of patient safety was reflected in a raft of policy developments, reports and widespread institutional and regulatory changes.^43^ For example, in 2001, a National Patient Safety Agency (NPSA) was created to act as a centre of learning and expertise for patient safety. An information system for adverse events, the National Reporting and Learning System (NRLS), was established in 2003, while a succession of regulatory agencies, most recently the independent Care Quality Commission (CQC) from 2009, were created to monitor and inspect the quality and safety of care in hospitals. At the ‘coalface’ of care in hospitals, the focus on patient safety became reflected in various clinical and managerial practices: the use of clinical audits, standards, guidelines and protocols; checklists and risk assessments; systems for reporting and investigating incidents; alerts on major risks; improvement campaigns and arrangements for staff to discuss the impact of delivering healthcare, such as Schwartz Rounds. Patient safety became an explicit right of patients in the NHS Constitution in 2009, gained ministerial oversight in 2019, and a Patient Safety Commissioner for England was appointed in 2022. In the sense that ‘patient safety’ now implies the need to *systematically* protect patients against avoidable harm (and especially, against errors by clinicians), it is only since 2000 patient harm has been considered in this way.

The emergence of patient safety thus represented more than a belated recognition by policymakers of the extent of patient harm. Patient safety solidified as a distinct way of thinking, which considered harm an emergent property of complex healthcare systems, rather than an unavoidable dimension of modern healthcare or the fault of individual clinicians. There was a movement of concern, from the actions and behaviours of individuals to the wider system in which they worked. This system could be designed and modified to reduce the risk of harm, and even, some hoped, eliminate avoidable harm altogether.^44^

The shape patient safety assumed after 2000, and its technical language, was therefore distinctly novel. Patient safety embodied the idea that action needed to be taken at the level of the health system and the state as well as the profession or clinician. The need to protect patient safety became embedded in systems of regulation, quality assurance and performance monitoring, designed to hold hospitals and professionals to account.^45^ Patient safety also crystallised as an academic discipline, with a discrete set of theories, tools and approaches to patient harm.^46^ Patient safety emerged as a policy goal in its own right, but one which could be related to the wider goal of ‘quality improvement’.^47^ For reasons I discuss later, however, the interest in healthcare quality did not straightforwardly convert into an interest in patient safety. The interests of quality improvement were wider, and clinicians could neglect a focus on adverse outcomes in favour of other measures.

All this begs the question: if ‘do no harm’ is at the centre of medicine, why was a systemic language of patient safety not employed before? One reason, as I argue below, was that the prevalence of adverse events was largely unknown until the millennium, when retrospective case-record reviews were conducted, and later, systemic reporting arrangements established.^48^ More fundamentally, however, were various barriers that dampened and silenced concerns, preventing the scale of patient harm from becoming more widely known, and inhibiting scientific research into error.^49^ It is to these I now turn.

## The Wall of Silence: Iatrogenic Injury and Error Before Patient Safety

A starting point to consider the emergence of patient safety as an explicit policy issue in the NHS is to consider the professional culture of caregivers. Clinicians’ attitudes towards mistakes, errors or omissions provide some of the most dramatic and visible clues for beliefs and values around patient harm and safety historically. The conditions of possibility for patient safety were shaped, in part, by how clinicians responded to the perennial problem of patient harm within the context of their professionalism; once the parameters of this professionalism changed, as happened in Britain in the 1990s and early 2000s, then a new, more all-encompassing notion of patient safety could come into being.

In relation to doctors, perhaps the greatest value shaping the attitude of the medical profession to patient harm was autonomy: the belief doctors have specialist skills and expertise that external groups (such as politicians, managers and other professionals) cannot scrutinise, and which gives doctors freedom to exercise individual judgement. The British medical profession originally won the right to regulate itself in the 1858 Medical Act, as part of what Margaret Stacey referred to as a ‘regulatory bargain’ between the profession and state.^50^ In exchange for controlling entry into the profession via the medical register, doctors implicitly promised to protect patients and provide safe and effective treatment.

The culture of medicine has long been described as ‘tribal’ and ‘club-like’. This culture was inculcated in doctors through medical education and training, and reinforced by patronage and elitism.^51^ At least in theory, as Dixon-Woods and colleagues argue, through this process all doctors were thought to be ‘sufficiently conditioned by norms acquired during socialisation … to ensure that all members would conduct themselves honourably’.^52^ In practice, the disciplinary processes of the profession could still be invoked, as seen in cases of doctors neglecting or assaulting their patients, disparaging colleagues or advertising inappropriately. However, this pattern of socialisation had important consequences for safety. First, it assumed all registered doctors had a certain level of competence and professional ‘conscience’. Second, the principles of professional autonomy and self-regulation meant only doctors could judge whether care was ‘safe’ or not.^53^

When the NHS was created in 1948, the professional autonomy and power of doctors was preserved. Until the introduction of ‘general management’ in the 1980s, hospital doctors were responsible for much of the health service’s administration. For instance, the MOH could exert little control over how doctors spent money, since doctors had discretion to provide whatever treatments they saw fit under their allocated budgets. Nurses, administrators and others were expected to defer to doctors’ expertise and authority.^54^ Professional codes specifically emphasised nurses’ subordinance to doctors. For example, the International Council of Nurses’ code of 1953 stated: ‘Nurses shall maintain the trust the public places on the doctor and other members of the health care team. Professional incompetence or lack of ethics on the part of one of these members shall be reported only to the competent authority’.^55^ The implicit threat was by raising concerns about doctors’ conduct (except through circumscribed channels), nurses could risk disciplinary action.

Under employment contracts from 1948, hospital doctors reaffirmed the ‘tribal’ culture that had dominated the profession since its inception, a culture satirised by authors such as George Bernard Shaw and A.J. Cronin (in *The Doctor’s Dilemma*, Shaw referred to the medical profession as a ‘conspiracy against the laity’).^56^ Despite significant changes in medical practice over the second half of the twentieth century (from the rise of group practice in primary care, to the increasing role of non-clinically trained managers from the 1980s and the crystallisation of ideas around informed consent and patient-centred care), this tribal culture could persist. Even in the 1990s, the doctors’ regulator, the General Medical Council (GMC), resembled a London gentleman’s club, with wood-panelled walls and plush leather seats.^57^ As late as 2001, the report into the Bristol Heart Scandal noted a ‘club culture’ among doctors at Bristol Royal Infirmary and the reluctance of managers—even those clinically trained—to criticise doctors. As Dr John Roylance, the Chief Executive of United Bristol Healthcare Trust, told the Bristol Inquiry, ‘only clinicians could identify defects in the performance of other clinicians’; the role of managers was ‘to provide and co-ordinate the facilities which would allow the consultants to exercise clinical freedom’.^58^ Throughout the twentieth century, this 'tribal', club-like culture of medicine was such that open discussion of error, particularly beyond the professional community, could often be discouraged. In 1990, the Deputy Editor of the *BMJ*, Tony Smith, wrote ‘all too often if the patient’s management has been less than optimal the reaction of those concerned is to say nothing’.^59^ Sir Donald Irvine, a GP and later President of the GMC (from 1995 to 2002), described ‘a professional culture in which the admission of error has been seen as difficult, the act of apologising as weak and defensive and the entitlement of people to know about the circumstances of error not readily conceded’.^60^ In a cultural milieu where technical knowledge and skill provided social capital, errors (such as surgery on the wrong limb) were often seen as doctors’ individual responsibility, and struck at the heart of their professional identity. Prevailing ideas of medical error assumed that given enough training or supervision, doctors’ errors could be eradicated completely.^61^

Errors, therefore, were not framed as systemic problems—as problems resulting from complex factors in the hospital, work and team environment—but individual problems, commonly expressed as professional incompetence or negligence. Punishment, for example, through professional censure, or rarely, through removal from the profession, was generally considered sufficient to discipline doctors and remove individual ‘bad apples’.^62^ So long as doctors (and managements) continued to think about error in this punitive and individualised way, then not only would error remain an open secret in healthcare, but also a wider language and approach to patient safety was not possible.

Managements are also tribal, and doctors speaking up about errors, avoidable deaths and other failures in hospitals could face severe repercussions. There have been many high-profile cases of whistleblowing doctors being victimised, dismissed from their jobs or even facing spurious referral to the GMC after raising concerns.^63^ Professional codes within medicine also discouraged doctors from publicly questioning the competence of colleagues. For example, the GMC’s guidance warned doctors ‘The council … regards as capable of amounting to serious professional misconduct … the deprecation by a doctor of the professional skill, knowledge, qualifications or services of another doctor or doctors’.^64^

The collegial model of self-regulation, together with the self-protective instincts of many hospital managements, enabled severe instances of patient harm and even acts of gross professional misconduct to be tolerated. In the extreme, as Dixon-Woods et al argue, ‘it was possible for some [doctors] to get away with murder (quite literally) or other repugnant behaviour over long periods’.^65^ It proved difficult for serious professional misconduct (SPM), the threshold for doctors to be removed from the medical register, to be proven at the GMC; even then, the GMC was slow to act on professional competence, as opposed to other aspects of professional behaviour.^66^ Statistics compiled by Russell Smith show alcohol, sexual and financial offences were by far the dominant concern of the GMC’s Professional Conduct Committee between 1858 and 1990, together accounting for more than 30 per cent of cases it considered.^67^ According to GMC guidance in 1979, the GMC was ‘not concerned with errors of diagnosis or treatment’; later guidance in 1983 clarified it was, but only if it raised a more fundamental question of SPM.^68^

The culture of medicine attracted significant criticism in the 1970s and 80s, notably from philosophers such as Ivan Illich (who re-popularised the psychiatric term ‘iatrogenesis’), and lawyers such as Ian Kennedy.^69^ The GMC’s disciplinary procedures also came into question, in part due to the rising influence of lay members on the Council (in particular, Jean Robinson) and growing patient/consumer representation in healthcare.^70^

The reasons why many doctors were so reluctant to criticise colleagues for mistakes also began to be explored by medical sociologists around this time. In addition to the consequences for their reputations, in large part, this stemmed from the uncertainties of clinical practice.^71^ Given the variability of bodies, symptoms and treatments and the limits of medical knowledge, medical failure was inevitable; it could happen to anyone. Charles Bosk’s famous ethnographic study of surgeons at an elite teaching hospital in the USA showed surgeons were willing to forgive colleagues for errors relating to technical skill or judgment, so long as they were not repeated.^72^ These were seen as ‘part of the job’ and even a training opportunity. However, attendings (equivalent to UK consultants) fiercely reprimanded colleagues for so-called normative errors, such as bad timekeeping. These errors were seen to be moral in character, to reflect badly on the individual in question, and by extension, their superiors.

The management of medical failure at an elite US teaching hospital may seem tangential to beliefs around patient harm and safety in the NHS. However, sociologists in the UK showed a similar propensity for doctors to forgive ‘routine’ error and suppress it, unless it was particularly serious or repeated. For example, in Rosenthal’s ethnography of British health practitioners in 1995, one Regional Director of Public Health remarked: ‘Doctors overwhelmingly cover up for each other on these matters. There are very strong group feelings that depend on colleague relationships. If the relationships are good, they cover up, if they are bad, they won’t ….’^73^ Presented with a ‘problem’ colleague, doctors often chose to deal with the problem informally, opting for a ‘terribly quiet chat’ with them, or stemming the referral of patients. In severe cases, doctors might refer colleagues to the ‘Three Wise Men’, medical panels that could consider support for doctors, and if necessary, disciplinary action.^74^ However, as this procedure evolved in the NHS, it was largely oriented to cases of ill-health and disability among doctors (including alcohol and drug addiction) rather than the prevention of ‘untoward incidents’. Indeed, in negotiations with the MOH about the procedure, senior representatives of the medical profession (the Joint Consultants’ Council) had consciously steered discussions away from the thorny issue of professional competence, which raised wider questions about patient safety.^75^ As this discussion highlights, a defining feature of the NHS’s response to patient harm before 2000 was it was largely considered a professional or clinical matter.^76^ While mechanisms existed to analyse and respond to adverse events, these often operated at a professional level, or the level of the medical/surgical specialty. At a managerial level, the NHS had few systems for learning from adverse events, and those existing were ineffective and poorly integrated.^77^

An example of a professional/specialist system of learning is the national confidential enquiries—essentially, national audits examining adverse outcomes in certain clinical areas.^78^ In 1952, a Confidential Enquiry for Maternal Deaths (CEMD) was established in England and Wales, following the precedent of earlier audits by local health boards from the 1920s. Further enquiries followed: for perioperative deaths (1988); stillbirths (1992); suicides (1992) and maternal and child health (2003). The main feature of these enquiries was that they relied (and continue to rely) on confidential reporting. Clinicians were encouraged to report deaths in their care (or within a certain period after discharge); these cases (or a sample) were professionally reviewed and the data used to inform clinical recommendations. While clinicians believed confidentiality encouraged reporting, and there is evidence clinical recommendations helped to improve outcomes in particular fields, such as obstetrics, it is unclear how the enquiries helped to promote patient safety across the wider NHS. Reporting was voluntary; coverage was limited; individual cases were not necessarily acted upon and there was no obligation for clinicians to implement the resulting recommendations.^79^

Another example of how doctors managed harm is the ‘mortality and morbidity’ (‘M & M’) conference. Following Codman’s work in the USA to study adverse outcomes, these meetings became common practice in many hospitals over the twentieth century in response to untoward or unexpected events. They allowed junior surgeons to raise issues as part of their training.^80^ However, M & M conferences were an example of ‘siloed’ learning: they were private affairs, managed by the medical community within hospitals, and knowledge gained was not usually disseminated further.^81^

Local medical audits—attempts to systematically evaluate medical quality—also offered few insights into adverse events. The Conservative government formalised doctors’ participation in audit in 1989. However, audit practices were variable: as Tony Smith warned, ‘The word audit is not some magic talisman that will change practice simply by repetition’.^82^ Only rarely did audit extend to the analysis of adverse events, as opposed to the effectiveness of interventions or opportunities for professional development.^83^ Despite being promoted by the Royal Colleges, many clinicians resisted audit as a managerial attack on their autonomy.^84^ As late as 1992, many doctors retained the view medical accidents should be solely investigated by professionals—that is, they wished to continue policing themselves.^85^ For these reasons, the managerial focus on quality in the 1980s and 1990s did not naturally translate into a concern with safety. Researchers implored the NHS to embrace a wider understanding of quality that included investigating adverse events.^86^ The systemic language of patient safety from 2000 reflected this broader understanding of quality.

Ultimately, because of doctors’ resistance to managerial scrutiny and the weakness of existing systems, little was known about the prevalence of adverse events in Britain until the early 2000s. The rate of medical negligence cases offered a clue, but they were a crude barometer, since so few cases proceeded to trial, and not all errors were negligent.^87^ Nor could much be learned from complaints. Despite medical opposition, a coordinated complaints procedure was mandated for NHS hospitals in 1985.^88^ However, health authorities did not collect, analyse or disaggregate complaints data in useful way for safety purposes. Various cultural barriers (such as patients’ reluctance to criticise doctors, and a ‘gratitude barrier’ revolving around freely accessible NHS care) also dissuaded many patients from complaining to begin with.^89^ It was not until 1981 the DHSS issued guidance to NHS hospitals on handling ‘clinical’ complaints, as opposed to ‘non-clinical’ complaints such as staff attitudes. Until 1996, the Health Service Commissioner (Ombudsman), which acted as a route of appeal for NHS complainants, was also prevented from investigating complaints relating to the ‘exercise of clinical judgement’. This effectively excluded most complaints which identified or alleged clinical error.^90^

Revealingly, most areas of NHS managerial activity before 2000 which explicitly related to safety corresponded with non-clinical activities or those associated indirectly with clinical practice. These included infection control arrangements, medicine safety, the safety of medical equipment and devices, radiological and laboratory safety and the health and safety of staff. In short, there was no systemic language of patient safety encompassing what Klein referred to as the ‘sacred realm of clinical autonomy’.^91^ In addition to doctors’ defence of their autonomy, and the professional consequences of speaking up about harm, perhaps the greatest factor preventing the scale of harm from becoming more widely recognised before 2000 was doctors’ fear of being sued. As a noted judge, Lord Denning, remarked in 1954—referencing *Macbeth*—‘an action for negligence against a doctor was like unto a dagger; his professional reputation was as dear to him (sic) as his body—perhaps more so’.^92^ In 1990, an anonymous doctor remarked to *The Guardian* how:

Patients trust their doctors. They have to, because their lives are in our hands. Sometimes we have to tell little white lies, just to preserve that trust. The lies are less blatant than they used to be, because patients are more informed, but it is still preferable to keep minor medical errors quiet to avoid damaging legal action.^93^

The importance of reputation and autonomy to doctors was reflected in the fact from 1948, doctors working exclusively within hospitals continued to indemnify themselves against medical negligence claims (by subscribing to defence organisations), despite being salaried NHS employees.^94^ Representatives of the profession lobbied the government for doctors to retain these subscriptions, in part because it was felt if health boards assumed full responsibility for defending doctors, then boards were encouraged to settle cases early rather than fight them.^95^ This gave doctors the chance to defend their reputations, but also meant they were exposed to the consequences of being sued.

Doctors’ fear of litigation is curious given the problems patients faced in securing compensation. Initiating a case against a doctor and/or health authority was not a decision to be taken lightly. Successful claims were comparatively rare. If cases failed, claimants were expected to pay the defence’s costs, which could be ruinous (some claimants were even forced to sell their homes). Legal aid was difficult to secure, since the Law Society (formed in 1825, and which originally administered legal aid) required a statement of medical opinion to determine worthy cases, and for the reasons explained, many doctors were unwilling to break ranks.^96^ Delays in cases were common—some stretched on for over a decade—and patients’ solicitors faced problems gaining access to relevant information, such as case notes. Unlike doctors, whose defence organisations existed since the nineteenth century, in Britain, there was no organisation to specifically act as the voice of patients in negligence cases (or patient safety more widely) until Action for Victims of Medical Accidents (AVMA) was established in 1982.^97^ The Patients Association, established in 1963, and the Community Health Councils (CHCs), since 1974, supported patients, for example, helping them to bring complaints. However, they had little legal expertise to help patients pursue claims, and most CHCs referred claimants directly to solicitors or AVMA.^98^

Given these problems, it is unsurprising that even in the 1990s, litigation could be depicted by those promoting tort reform (such as the MP, Rosie Barnes) as a ‘cruel lottery’.^99^ Injured patients, facing life-changing legal costs, often chose to settle their cases early rather than pursuing them further. Many did not even attempt to seek compensation—often, they did not even know they had a case, since defence solicitors could advise doctors or health authorities not to apologise, lest they give claimants the impression their case had merit.^100^ Despite claimants’ difficulties, fear of litigation kept many doctors in the NHS silent about mistakes. A survey on the impact of litigation on British clinicians in 1994 revealed anger, distress, shame and victimisation were all common reactions to being sued, and clinicians could feel isolated from colleagues and management.^101^

## Clinical Risk Management: A Moment of Departure For Patient Safety?

Whatever the chances of individual claims succeeding, the overall level of litigation in the 1980s and 90s fuelled doctors’ concerns about being sued. Over the 1980s, partly because of improved support and information for patients by groups such as AVMA, the rate of claims in the NHS increased significantly. In the Oxford region alone, the frequency of claims increased fivefold over the 1980s, and the cost of meeting successful claims increased 250 per cent.^102^ Organisations representing doctors, such as the Medical Defence Union (MDU) and British Medical Association, feared the import of an American-style culture of litigation, and ‘defensive medicine’—doctors refusing to undertake certain procedures, or even leaving certain ‘high-risk’ specialties such as obstetrics altogether. An MDU report in 1988 blamed rising litigiousness on ‘the patient’s unrealistic expectation of the healing powers of his or her doctor. Whenever a doctor or dentist failed to achieve a perfect result, today’s patient is likely to consider recourse to law’.^103^ The irony in this statement was that by marginalising error and keeping silent about mistakes, doctors themselves had moulded the image of perfectibility they now resisted.

The media coverage around litigation was ambivalent. On the one hand, newspapers recounted tales of medical ‘blunder’ which were clearly at odds with the popular trope of the heroic doctor and self-sacrificing nurse.^104^ Current affairs programmes such as Thames Television’s *Medical Mistakes—Who Pays the Price?* (1983) recounted patients’ experiences of the legal process, and gave potential claimants advice about how to deal with the NHS.^105^ On the other hand, newspapers reported with alarm the growing sums awarded to victims of medical negligence (especially the considerable sums awarded to infant victims of brain damage, often to provide for life-long care).^106^ Massive awards were portrayed as a burden on the NHS, and fuelled arguments that the legal system was out of control.

In response to increasing litigation, doctors’ subscriptions to defence societies soared. By 1988, the average subscription was £1080, an increase of 2,700 per cent over ten years. Between 1986 and 1990, the cost of doctors’ subscriptions quadrupled.^107^ Doctors lobbied for a no-fault system of compensation which would automatically compensate injured patients rather than having them prove negligence in court. Based on the experiences of schemes in Sweden and New Zealand, doctors supported a private bill by Barnes on no-fault compensation in 1991, however, this failed since the government believed it would not reduce costs overall.^108^

The rising cost of defence society subscriptions threatened to further complicate NHS pay negotiations.^109^ In January 1990, therefore, the government introduced NHS indemnity. This placed the burden for meeting the costs of litigation onto NHS Trusts; no longer would hospital doctors have to indemnify themselves.

The consequences for patient safety were significant. The newly created NHS Trusts had additional incentive to reduce clinical errors. Over the 1990s, the discipline of clinical risk management (CRM) encouraged hospitals to introduce systems for managing the risk of claims, such as systems for staff to report incidents, and procedures for investigating accidents and complaints.^110^ Dedicated claims and clinical risk managers began to be employed. While the primary motivation behind CRM was financial, adherents (including researchers and legal firms) stressed its benefits for safety, preventing new incidents rather than reacting to those that had already occurred.^111^ Private companies launched software (such as Datix in 1986), which continue to be used in the NHS to log patient safety incidents. Around this time, the Medical Protection Society funded some of the earliest research on medical accidents in Britain, exploring among other things, the causes of accidents, the attitude of doctors and the psychological effect of accidents on patients.^112^

By 1994, the cost of settling claims in the NHS reached an estimated £75 million a year.^113^ This pressure encouraged further organisational developments, such as the Clinical Negligence Scheme for Trusts (CNST). Managed by the NHS Litigation Authority (now NHS Resolution), the scheme established a central fund by which NHS Trusts could pool their risks. It also introduced further incentives for Trusts to evolve risk management. In exchange for discounted premiums and less frequent auditing, Trusts were encouraged to develop more robust reporting systems, risk management policies and other mechanisms.^114^ In this way, CRM provided a managerial foundation for many of the practices and procedures integral to patient safety.

## Regulatory Crisis

The collegial model of self-regulation, and with it, the culture of silence around patient harm, came under intense political and media scrutiny in the 1990s. Poorly performing and incompetent doctors had long been of media interest. Scandals revolving around hospital standards in the NHS were also far from new (from the late 1960s to the early 80s, in particular, inquiries had exposed appalling standards of care in long-stay hospitals).^115^ However, the sheer frequency of scandals relating to the safety of clinical care (more specifically) in the 1990s raised damaging questions among politicians, the media and patient groups about the ability of doctors and the wider NHS to protect patients. The adverse publicity these scandals attracted encouraged the Labour government from 1997 to extend new forms of regulatory oversight over healthcare.

There was the case of Rodney Ledward, who allegedly styled himself ‘the fastest gynaecologist in the south-east’ after performing seven hysterectomies in under four hours. He was struck off after a series of errors in Kent.^116^ There was Richard Neale, another gynaecologist, who had been struck off the medical register in Canada following the deaths of two patients, yet was able to join the medical register in Britain (Neale was struck off in 2000 for various offences, including lying about his qualifications). There were various high-profile cases of sexual assault. The conviction of the GP Harold Shipman for murdering fifteen of his patients—and who was responsible for potentially hundreds more deaths—was another pivotal moment, calling into question the trusting relationship between doctor and patient. All these scandals erupted in the space of a few years and were accompanied by intense media coverage and campaigning by patient and victim support groups.^117^

In addition, there was the scandal around children’s heart surgery at Bristol. The ‘Bristol case’ was different, revolving around issues of poor clinical performance (high infant mortality) relative to other specialist cardiac units. Unlike errors in ‘routine’ surgery, paediatric cardiac surgery at Bristol was considered high risk and a certain degree of mortality was expected. Nevertheless, media attention focused on how long it took the mortality rate at Bristol to be acted upon, managerial inertia, and how it took a whistleblower (the anaesthetist Steve Bolsin) to raise concerns.

**Fig. 1:**
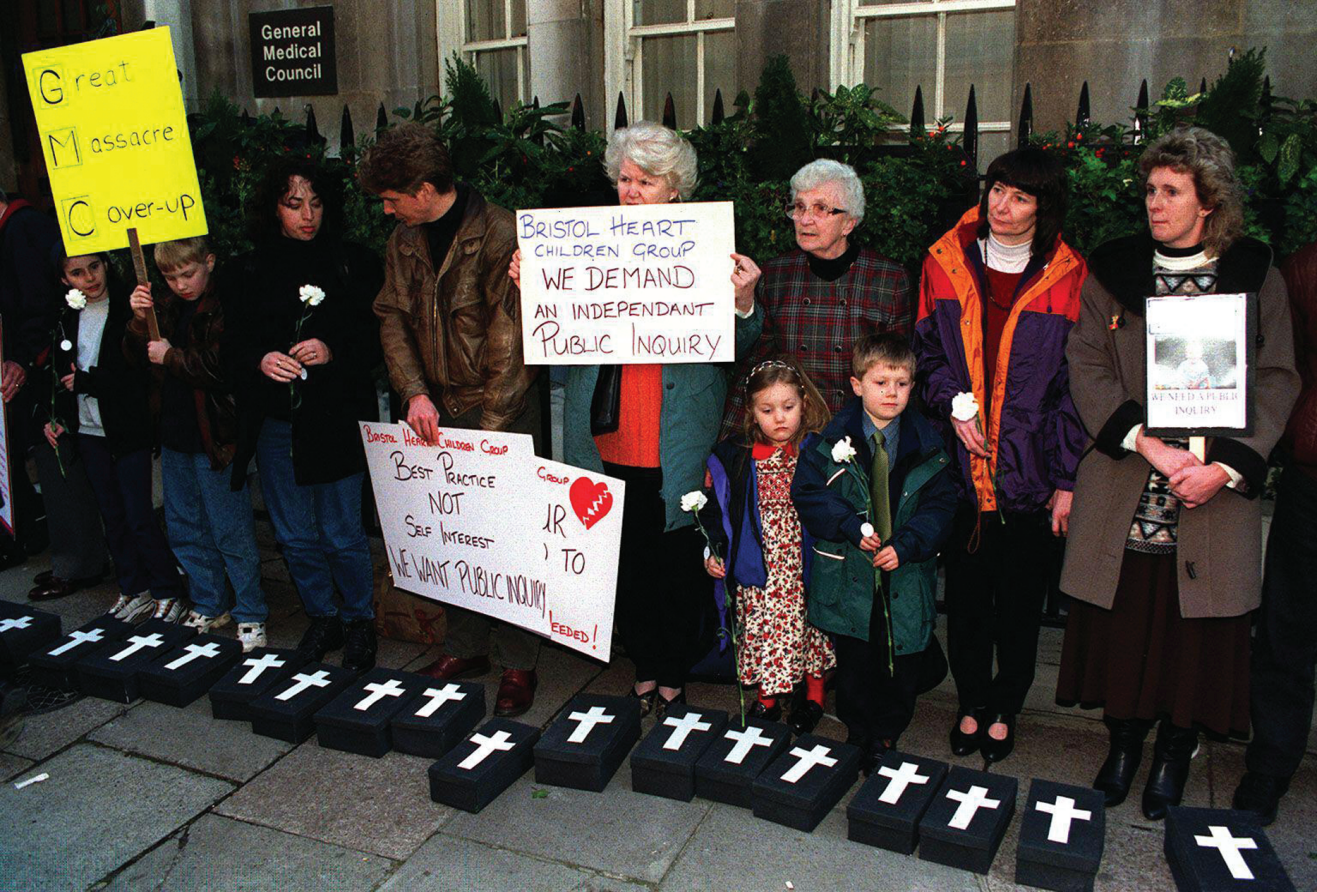
Demonstration outside GMC by the Bristol Heart Children Action Group, 18 February 1998. Photographer: Michael Stephens. Source: PA Images/Alamy Stock Photo. Used under licence

The Bristol case was highly emotive, involving the care of vulnerable infants on a part of the body long seen as the site of the emotions.^118^ This symbolism and imagery, together with the dramatic way the scandal unfolded in the media and the suggestions of conspiracy and cover-up, underpinned public discourse which portrayed the medical profession as secretive, insular and self-protecting. In February 1998, families of children who died or were injured at Bristol picketed the headquarters of the GMC, calling for a public inquiry. Carrying lighted candles, they laid on the ground fifty small black coffins, one for every child thought to have died. Above their heads, a placard read: ‘GMC: Great Massacre Cover-Up’ (Figure 1).^119^ The culture and regulation of medicine fell into disrepute. As *The Independent* put it in 2000: ‘Is there something rotten at the heart of the medical profession? We once trusted our doctors unquestioningly. But now week after week we seem to read headlines about doctors who are incompetent, unprofessional, or worse. And patients are losing confidence’.^120^

By striking off two of the Bristol doctors, including the Chief Executive of the Bristol Trust, the GMC signalled failing to act on poor performance—for example, by neglecting to update knowledge and skills—could now be interpreted as SPM. However, the GMC’s verdict had wider consequences for patient safety and the relationship between doctors, patients and the state. For the government and critics of medical self-regulation, it suggested the need for a new model of regulation, accountability and safety. As the *BMJ’s* editor, Richard Smith, remarked following the verdict (referencing Yeats), ‘all changed, changed utterly’.^121^

These various scandals precipitated a ‘regulatory crisis’ in British medicine.^122^ It is against the backdrop of this crisis we must return to Donaldson’s report *AOWAM* in 2000 and the ‘tragedies’ it addressed. New Labour inherited the issue of incompetent and poorly performing doctors when it assumed power in 1997. Its NHS white paper announced the government’s intention to ‘modernise’ and improve the quality of care in the NHS, continuing a major theme of Conservative health policy. However, it went further. Among other things, Labour sought ‘to strengthen the existing systems of professional self-regulation’ by making them ‘open, responsive and publicly accountable’.^123^ The concept of clinical governance, introduced by the white paper *A First Class Service* in 1998, married concerns about provider accountability and quality improvement with mechanisms to facilitate good practice.^124^ These included clinical standards, as laid down by the new National Institute for Clinical Excellence (NICE); National Service Frameworks, which defined standards in specific areas of clinical activity and a new regulator, the Commission for Health Improvement, to monitor hospital performance.^125^

Hence, the framework of patient safety emerging in the NHS from 2000 formed a natural continuation of the clinical governance agenda. The new framework repudiated the idea patient safety could be dealt with by doctors alone. What was needed was a wider approach in which healthcare professionals and managers collectively accounted for safety.^126^ The new processes developing in the NHS were not restricted to health professionals, but were system-wide.

It is at this point scientific research on the causes of accidents in complex industrial systems began to influence the political dialogue. The field of industrial safety science had developed rapidly following a slew of major disasters around the world in the 1970s and 1980s: the partial meltdown at the Three Mile Island nuclear power plant in the United States (1979); the Bhopal chemical disaster in India (1984); the Challenger space shuttle disaster (1986) and the Chernobyl Disaster in Ukraine (1986). Such disasters—and the detailed investigations into them—spawned new scientific approaches to the issue of ‘human error’ in complex industrial systems, as scientific attention moved to the organisational and managerial factors that promoted safety (or increased the risk of accidents). Around this time, the idea that organisations could possess a distinct ‘safety culture’ also began to crystallise.^127^ Britain in particular experienced a series of industrial and public safety disasters over this period that challenged how scientists and regulators approached the concept of risk: from the explosion at the Nypro chemical plant in Flixborough (1974), to the deadly fire at King’s Cross Underground Station (1987) and the devastating crush at Hillsborough Football Stadium (1989). Regulatory attention increasingly focused on how safety could be secured proactively as part of the everyday management of operations, and managed through formal techniques of risk assessment.^128^ The need for such techniques became increasingly apparent to regulators, as the rising complexity of industrial operations—which arguably included healthcare—was seen to merit greater focus on work processes and systems.

In this scientific context, models of accident causation in complex industrial systems were particularly attractive to those reform-minded scientists, clinicians and managers keen to promote safety in healthcare. In particular, the ‘Swiss cheese’ model, devised by the British psychologist James Reason, viewed ‘active failures’ by individuals (e.g., clinical errors), to be precipitated by ‘latent conditions’, problems in the work environment and systems ‘upstream’ in organisations.^129^ By the mid-1990s, such theories, initially popularised in industry, began to be adapted in healthcare through a series of conferences in the USA.^130^ Reason attended the influential Annenberg Conference in 1996, and was later a member of the Expert Group on Learning from Experience.

The Expert Group was commissioned by the Health Minister, Baroness Hayman in 1999. In addition to Donaldson and Reason, members included the Health Service Commissioner (Michael Buckley), and the psychologist Charles Vincent, who had conducted research on medical accidents and clinical risk since the 1980s.^131^ The committee appears to have been convened partly in response to lobbying by patient groups and scrutiny of the NHS’s managerial systems. In April 1999, the House of Commons Health Committee investigated how the NHS responded to adverse clinical incidents and outcomes, highlighting the defensiveness of doctors and health authorities to complaints, the ‘culture of blame’ preventing doctors and nurses from reporting incidents, and the patchwork of systems and processes undermining a coherent response.^132^

Among those testifying were Will Powell, representing the Bereaved Parents’ Group. Powell had lost his ten-year-old son, Robbie, from Addison’s disease, amid cover-up and maladministration by doctors, health authorities and the police. In a High Court ruling in 1996, Powell’s case had demonstrated the absence of a ‘free-standing’ legal duty of candour on health professionals to be open and honest with families following a relative’s death.^133^ In a move DOH officials thought to be unusual, members of the Health Committee travelled to Leeds to interview NHS staff and the patients of poorly performing surgeons.^134^ Political scrutiny and lobbying by patient groups thus encouraged the DOH to establish the Expert Group to generate answers to these systemic problems.^135^

The Expert Group’s report, *AOWAM*, endorsed the Health Committee’s view of a fragmented and ineffective system of learning from adverse events. Building on the clinical governance framework, *AOWAM* recommended new systems be set up nationally for the NHS to report and learn from adverse events. Following a subsequent report by the DOH, *Building a Safer NHS for Patients*, the National Patient Safety Agency (NPSA) was established, along with a voluntary system for frontline staff to report incidents and near-misses, the National Reporting and Learning System (NRLS).^136^

In this way, the emergence of patient safety in the NHS reflected an expansion of state regulation of medical care, modifying the ‘regulatory bargain’ between state and medical profession.^137^ Further, it reflected the growth of a culture of audit, performance monitoring and accountability, located across the public sector since the 1980s and linked with new notions of public management, but enshrined in the NHS since the reforms of the Conservative government in the late 1980s (for instance, the *Patient’s Charter* and the formalisation of clinical audit).^138^ The autonomy of the medical profession had already begun to erode in response to ‘scientific-bureaucratic’ medicine: the establishment of an increasingly managerial system of healthcare, reflected in the rise of general management in the 1980s and concerns about ‘evidence-based medicine’ and cost-effectiveness.^139^ However, the scrutiny accompanying healthcare scandals provided opportunity for politicians, patient groups and reform-minded doctors and managers (such as Donaldson) to challenge engrained beliefs and practices. The moment of patient safety reflected the fact that adverse events were no longer perceived be the exclusive concern of clinicians, to be shielded from wider scrutiny. Adverse events were now the concern of the healthcare system as a whole, and were increasingly thought to manifest from the healthcare system as a whole.

## Conclusion

Over the last two decades, fundamental changes have occurred in the relationship between the medical profession, patients and the state. The problem of patient harm is ancient, but since the emergence of patient safety around 2000, it has been reconceptualised. No longer is patient harm considered an ‘inevitable’ by-product of healthcare, something which only doctors have the expertise and power to address. Nor is clinical error seen to be the sole responsibility of individuals. Since the moment of patient safety, to err is increasingly seen to be human.^140^ Error and harm are thought to occur within, and to be caused by, wider systems of healthcare delivery. These systems can be designed, monitored and regulated to reduce the risk of error and harm. As part of this movement, insights from safety in other industries—notably aviation—have been applied to healthcare. The increasing use of techniques such as simulation training (as promoted by Donaldson),^141^ the systems analysis of critical incidents and attention to the human factors which shape safe and unsafe care are all evidence of such a borrowing. What is clear is responsibility for preventing and ameliorating harm has moved beyond clinicians, to encompass managers, regulators, policymakers, scientists and others.^142^

This article has explored the cultural changes in and around the NHS that shaped the ‘moment of patient safety’—this period around 2000 when an explicit discourse of patient safety emerged, and when patient safety became the object of political scrutiny, management and regulation. I have highlighted longer-running themes in the history of the NHS—and the culture of the medical profession—that prevented a systemic language of patient safety from emerging before this time, as well as developments in the 1980s and 90s that elevated patient safety as a policy issue. These include the rapid increase in the cost and extent of clinical negligence claims, and a succession of healthcare scandals in the 1990s that damaged public and political trust in medical self-regulation.

The report of the public inquiry into healthcare failures at Mid Staffordshire NHS Foundation Trust in 2013 offers an incisive bookend to this account of patient safety. Here, the shocking instances of abuse and neglect exposed by Julie Bailey and her campaign group Cure the NHS were seen to be facilitated by an ineffective, uncaring and unaccountable *system* of monitoring and regulation. Neither the Trust management nor the many agencies established since 2000 to monitor quality and safety at the Trust placed the interests of patients first, and all failed to act upon the warning signs of patient harm. Indeed, the chair of the public inquiry, Robert Francis QC, suggested these agencies operated within a new culture, ‘doing the system’s business’.^143^ While the moment of patient safety affirmed the NHS as a whole was responsible for patient harm, recurring healthcare failures since 2000 (most recently, around maternity services) suggest the NHS still has a long way to go to embody a genuine ethos of patient safety, rather than treating it as a performative or box-ticking exercise. The need to promote a ‘just culture’ in the NHS where errors are readily admitted, and where staff exercise accountability, has been a continual theme of reports over the last twenty years.^144^ This suggests much of the answer to patient harm may lie not in systems or processes—the focus of so many patient safety efforts since 2000—but attending to the more complex human and affective dimensions of care, so lost in the bureaucratic focus on performance since the millennium.

## Footnotes

1 Department of Health, *An Organisation with a Memory: Report of an Expert Group on Learning from Adverse Events in the NHS* (London: TSO, 2000).

2 Liam Donaldson, ‘An Organisation with a Memory’, *Clinical Medicine*, 2002, 2, 452–57.

3 Mary Dixon-Woods, Karen Yeung, and Charles L. Bosk, ‘Why is UK Medicine No Longer a Self-Regulating Profession? The Role of Scandals Involving “Bad Apple” Doctors’, *Social Science & Medicine*, 2011, 73, 1452–59.

4 DOH, *Organisation with a Memory*, v.

5 Ibid., viii, xii; Charles Vincent, Graham Neale, and Maria Woloshynowych, ‘Adverse Events in British Hospitals: Preliminary Retrospective Record Review’, *BMJ*, 2001, 322, 517–19.

6 Judy Jones, ‘A Catalogue of Blunders’, *The Observer*, 31 October 1993.

7 Charles Vincent, *Patient Safety*, 1st edn (Edinburgh: Elsevier Churchill Livingstone, 2006), 14.

8 In 1863, for instance, Nightingale wrote ‘It may seem a strange principle to enunciate as the very first requirement in a Hospital that it should do the sick no harm’. Florence Nightingale, *Notes on Hospitals*, 3^rd^ edn (London: Longman, Green, Longman, Roberts, and Green, 1863), iii.

9 Ian A. Burney, *Bodies of Evidence: Medicine and the Politics of the English Inquest, 1830–1926* (Baltimore: Johns Hopkins University Press, 1999).

10 Lucian L. Leape, ‘Error in Medicine’, *JAMA*, 1994, 272, 1851–57.

11 Oliver Quick, *Regulating Patient Safety: The End of Professional Dominance?* (Cambridge: Cambridge University Press, 2017).

12 Although, see Ian Leistikow and Frank Huisman, ‘The Role of the Patient in Patient Safety: What Can We Learn from Healthcare’s History?’, *Journal of Patient Safety and Risk Management*, 2018, 23, 139–41.

13 Virginia A. Sharpe and A.I. Faden, *Medical Harm: Historical, Conceptual, and Ethical Dimensions of Iatrogenic Illness* (Cambridge: Cambridge University Press, 1998); Thomas Schlich and Ulrich Tröhler, eds, *The Risks of Medical Innovation: Risk Perception and Assessment in Historical Context* (London: Routledge, 2006).

14 Jonathan Reinarz and Rebecca Wynter, eds, *Complaints, Controversies and Grievances in Medicine: Historical and Social Science Perspectives*, Routledge Studies in the Sociology of Health and Illness (London: Routledge, 2015); Alex Mold, *Making the Patient-Consumer: Patient Organisations and Health Consumerism in Britain* (Manchester: Manchester University Press, 2015); Linda Mulcahy, *Disputing Doctors: The Socio-Legal Dynamics of Complaints about Medical Care* (Maidenhead: Open University Press, 2003).

15 Duncan Wilson, *The Making of British Bioethics* (Manchester: Manchester University Press, 2014).

16 Kim Price, *Medical Negligence in Victorian Britain: The Crisis of Care under the English Poor Law, c.1834-1900* (London: Bloomsbury Academic, 2015).

17 Mold, *Making the Patient-Consumer*.

18 Quick, *Regulating Patient Safety*, 37 and passim.

19 Mold, *Making the Patient-Consumer*; Reinarz and Wynter, *Complaints, Controversies and Grievances in Medicine*.

20 Rudolf Klein and Ann Howlett, *Complaints Against Doctors: A Study in Professional Accountability* (London: C. Knight, 1973), 105–6.

21 Mulcahy, *Disputing Doctors*.

22 Lucian L. Leape, *Making Healthcare Safe: The Story of the Patient Safety Movement* (Cham: Springer International Publishing, 2021); Robert L. Wears and Kathleen M. Sutcliffe. *Still Not Safe: Patient Safety and the Middle-Managing of American Medicine* (New York: Oxford University Press, 2020).

23 Troyen A. Brennan et al., ‘Incidence of Adverse Events and Negligence in Hospitalized Patients’, *New England Journal of Medicine*, 1991, 324, 370–76; Lucian L. Leape et al., ‘The Nature of Adverse Events in Hospitalized Patients’, *New England Journal of Medicine*, 1991, 324, 377–84.

24 Wears and Sutcliffe, *Still Not Safe*; Linda T. Kohn, Janet M. Corrigan, and Molla S. Donaldson, eds, *To Err Is Human: Building a Safer Health System* (Washington, DC: National Academies Press, 2000).

25 Leape, ‘Error in Medicine’, 1851.

26 Wears and Sutcliffe, *Still Not Safe*.

27 See Wears and Sutcliffe, *Still Not Safe*; J.T. Reason, *Human Error* (Cambridge: Cambridge University Press, 1990); J.T. Reason, *Managing the Risks of Organizational Accidents* (Aldershot: Ashgate, 1997). In Britain, such insights have been promoted by individuals such as Liam Donaldson, and by organisations such as the Clinical Human Factors Group (CHFG), established by the airline pilot Martin Bromiley in 2007 (see https://chfg.org/about-chfg/, accessed 1 December 2022). Aviation’s influence is also reflected in the Healthcare Safety Investigation Branch (HSIB), established in 2017. Modelled on accident investigation in aviation, it investigates patient safety incidents on a technical level, without apportioning liability or blame.

28 For example, the World Health Organisation (WHO) passed its first resolution on patient safety in 2002, while Donaldson played a key role in the development of the World Alliance for Patient Safety, established in 2004. Leape, *Making Healthcare Safe*, 215–16.

29 Wears and Sutcliffe, *Still Not Safe*; Quick, *Regulating Patient Safety*; Stephen Harrison and Bruce Wood, ‘Scientific-Bureaucratic Medicine and UK Health Policy’, *Review of Policy Research*, 2000, 17, 25–42; Mirko Noordegraaf, ‘From “Pure” to “Hybrid” Professionalism: Present-Day Professionalism in Ambiguous Public Domains’, *Administration & Society*, 2007, 39, 761–85.

30 Indeed, the synchronous flowering of interest in patient safety on either side of the Atlantic suggests the importance of a cultural explanation for the rise of this interest in addition to transnational intellectual linkages.

31 Wears and Sutcliffe, *Still Not Safe*.

32 Virginia A. Sharpe and A.I. Faden, *Medical Harm: Historical, Conceptual, and Ethical Dimensions of Iatrogenic Illness* (Cambridge: Cambridge University Press, 1998).

33 Ibid.

34 Roy Porter, *The Greatest Benefit to Mankind: A Medical History of Humanity from Antiquity to the Present* (London: HarperCollins, 1997), 369–75.

35 Wears and Sutcliffe, *Still Not Safe*, 18; Quick, *Regulating Patient Safety*.

36 Schlich and Tröhler, *Risks of Medical Innovation.*

37 R.H. Moser, ‘Diseases of Medical Progress’, *The New England Journal of Medicine*, 1956, 255, 606–14; D.P. Barr, ‘Hazards of Modern Diagnosis and Therapy: The Price We Pay’, *Journal of the American Medical Association*, 1955, 159, 1452–56.

38 The National Archives, London (TNA), MH 99/168, memo, Heasman to Davies, 19 December 1962. See also W.E. Snell, ‘Accidents to Patients in Hospital’, *The Lancet*, 1956, 268, 1202–4. In contrast, falls are now the most widely reported patient safety incident, with over 250,000 reported annually in England and Wales. Rob Morris and Shelagh O’Riordan, ‘Prevention of Falls in Hospital’, *Clinical Medicine*, 2017, 17, 360–62.

39 TNA JA 440/6/2, letter to J.R. Rose, 27 September 1985.

40 London Metropolitan Archives, A/KE/I/01/69/004 and 014, ‘Safety in hospitals’.

41 John Abraham and Courtney Davis, ‘Testing Times: The Emergence of the Practolol Disaster and its Challenge to British Drug Regulation in the Modern Period’, *Social History of Medicine*, 2006, 19, 127–47.

42 Wears and Sutcliffe, *Still Not Safe;* see the Google nGram on p. 17.

43 See National Audit Office, *A Safer Place for Patients: Learning to Improve Patient Safety* (London: TSO, 2005); DOH, *Building a Safer NHS for Patients: Implementing An Organisation With A Memory* (London: TSO, 2001); DOH, *Safety First: A Report for Patients, Clinicians and Healthcare Managers* (London: Department of Health, 2006); DOH, *Good Doctors, Safer Patients: A Report by the Chief Medical Officer* (London: Department of Health, 2006).

44 Leape, ‘Error in Medicine’; Linda Emanuel et al., ‘What Exactly is Patient Safety?’, *Journal of Medical Regulation*, 2009, 95, 13–24.

45 Fundamentally, patient safety became part of the wider ‘audit culture’ that spread across the public and private sectors from the 1980s. Quick, *Regulating Patient Safety*; Michael Power, *The Audit Society: Rituals of Verification* (Oxford: Oxford University Press, 1997).

46 Emanuel et al., ‘What Exactly Is Patient Safety?’.

47 Charles Vincent, ‘Risk, Safety, and the Dark Side of Quality: Improving Quality in Health Care Should Include Removing the Causes of Harm’, *BMJ*, 1997, 314, 1775–76; Donald M. Berwick, ‘Continuous Improvement as an Ideal in Health Care’, *New England Journal of Medicine*, 1989, 320, 53–56; Martin D. Moore, *Managing Diabetes, Managing Medicine: Chronic Disease and Clinical Bureaucracy in Post-War Britain* (Manchester: Manchester University Press, 2019).

48 Vincent, Neale, and Woloshynowych, ‘Adverse Events in British Hospitals’.

49 C.A. Vincent, ‘Research into Medical Accidents: A Case of Negligence?’, *BMJ*, 1989, 299,1150–53.

50 Margaret Stacey, *Regulating British Medicine: The General Medical Council* (Chichester: John Wiley, 1992); Meg Stacey, ‘The General Medical Council and Professional Self-Regulation’, in D. Gladstone et al., eds, *Regulating Doctors* (London: Institute for the Study of Civil Society, 2000).

51 Eliot Freidson, *Profession of Medicine: A Study of the Sociology of Applied Knowledge* (Chicago: University of Chicago Press, 1988); Donald Irvine, *The Doctors’ Tale: Professionalism and Public Trust* (Abingdon: Radcliffe Medical Press, 2003).

52 Dixon-Woods, Yeung, and Bosk, ‘Why is UK Medicine’, 1455.

53 Ibid. These attitudes extended to legal practices. According to the prevailing definition of medical negligence (*Bolam v. Friern Hospital Management Committee*, 1957), doctors themselves defined the boundaries of normal and negligent practice. Arnold Simanowitz, *The Man Under the Clapham Omnibus: The Search for Patient Safety and Justice* (Brighton: Pen Press, 2013).

54 Rudolf Klein, *The New Politics of the NHS: From Creation to Reinvention*, 7^th^ edn (London: Radcliffe Publishing, 2013).

55 Taka Oguisso et al., ‘First International Code of Ethics for Nurses’, *Texto & Contexto Enfermagem* 2019, 28, e20180140.

56 George Bernard Shaw, *The Doctor’s Dilemma* (Harmondsworth: Penguin, 1971 [1913]); A.J. Cronin, *The Citadel* (Basingstoke: Bello, 2013 [1937]).

57 Irvine, *The Doctor’s Tale*.

58 DOH, *Learning from Bristol: The Report of the Public Inquiry into Children’s Heart Surgery at Bristol Royal Infirmary, 1984-1995*, Cm 5207(I) (London: TSO, 2001), 78. Similar sentiments were expressed at earlier inquiries. For example, following accusations of poor care at South Ockenden Hospital in 1974, an inquiry noted that the administrators of the hospital ‘distrusted the evidence of their own eyes.’ Martin, *Hospitals in Trouble*, 25.

59 Tony Smith, ‘Doctors Should Admit Their Mistakes’, *International Journal of Risk and Safety in Medicine*, 1990, 1, 45–47, 46.

60 Irvine, *The Doctor’s Tale*, 25.

61 Leape, ‘Error in Medicine’.

62 Ibid.; Dixon-Woods, Yeung, and Bosk, ‘Why is UK Medicine’.

63 See, for example, Peter Duffy, *Whistle in the Wind: Life, Death, Detriment and Dismissal in the NHS. A Whistleblower’s Story* (Independently Published, 2019).

64 General Medical Council (GMC), *Professional Conduct and Discipline* (London: GMC, 1979), 13; Jean Robinson, ‘The Price of Deceit’, in Marilynn M. Rosenthal, Linda Mulcahy, and Sally M. Lloyd-Bostock, eds, *Medical Mishaps: Pieces of the Puzzle* (Buckingham: Open University Press, 1999), 246–56.

65 Dixon-Woods, Yeung, and Bosk, ‘Why is UK Medicine’.

66 Robinson, ‘The Price of Deceit’.

67 Russell G. Smith, *Medical Discipline: The Professional Conduct Jurisdiction of the General Medical Council, 1858-1990* (Oxford: Clarendon Press, 1994); Quick, *Regulating Patient Safety*.

68 GMC, *Professional Misconduct and Discipline*; 9; GMC, *Professional Conduct and Discipline: Fitness to Practise* (London: GMC, 1983), 10; Quick, *Regulating Patient Safety*; Margaret Stacey, ‘A Sociologist Looks at the GMC’, *The Lancet*, 1989, 333, 713–14.

69 Ivan Illich, *Medical Nemesis: The Expropriation of Health* (London: Calder and Boyars, 1975); Samuel Gorovitz and Alasdair MacIntyre, ‘Toward a Theory of Medical Fallibility’, *The Hastings Center Report*, 1975, 5, 13–23; Ian Kennedy, *The Unmasking of Medicine* (London: Allen & Unwin, 1981); Neil McIntyre and Karl Popper, ‘The Critical Attitude in Medicine: The Need for a New Ethics’, *British Medical Journal*, 1983, 287, 1919–23.

70 See Robinson, ‘The Price of Deceit’; Jean Robinson, *A Patient Voice at the GMC: A Lay Member’s View of the General Medical Council* (Health Rights,1988); Mold, *Making the Patient-Consumer*; Mark Davies, *Medical Self-Regulation: Crisis and Change* (Ashgate Publishing, Ltd., 2007); Richard Smith, ‘Profile of the GMC: Discipline I: The Hordes at the Gates’, *BMJ*, 1989, 298, 1502–5.

71 Renée C. Fox, ‘The Evolution of Medical Uncertainty’, *The Milbank Memorial Fund Quarterly. Health and Society*, 1980, 58, 1–49.

72 Charles L. Bosk, *Forgive and Remember: Managing Medical Failure* (Chicago; London: University of Chicago Press, 1979).

73 Marilynn M. Rosenthal, *The Incompetent Doctor: Behind Closed Doors* (Buckingham: Open University Press, 1995), 26.

74 Ibid.

75 See TNA 99/165, ‘National Health Service. Prevention of Harm to Patients Resulting from Physical or Mental Disability of Hospital Medical or Dental Staff. H.M.(60) 45’.

76 Quick, *Regulating Patient Safety*, 3.

77 DOH, *Organisation with a Memory*.

78 S.M. Yentis, ‘From CEMD to CEMACH to CMACE To…? Where Now for the Confidential Enquiries into Maternal Deaths?’, *Anaesthesia*, 2011, 66, 859–60; A.M. Weindling, ‘The Confidential Enquiry into Maternal and Child Health (CEMACH)’, *Archives of Disease in Childhood*, 2003, 88, 1034–37; Aniela Angelow and Nick Black, ‘The Use and Impact of National Confidential Enquiries in High-Income Countries’, *BMJ Quality & Safety*, 2011, 20, 38–45.

79 Kieran Walshe, ‘Medical Accidents in the UK: A Wasted Opportunity for Improvement?’, in Marilynn M. Rosenthal, Linda Mulcahy, and Sally M. Lloyd-Bostock, eds, *Medical Mishaps: Pieces of the Puzzle*, 59–73.

80 Jay D. Orlander, Thomas W. Barber, and B. Graeme Fincke, ‘The Morbidity and Mortality Conference: The Delicate Nature of Learning from Error’, *Academic Medicine*, 2002, 77, 10, 1001–6.

81 Vincent et al, ‘Medical Accidents’; Juliet Higginson, Rhiannon Walters, and Naomi Fulop, ‘Mortality and Morbidity Meetings: An Untapped Resource for Improving the Governance of Patient Safety?’, *BMJ Quality & Safety*, 2012, 21, 576–85.

82 T. Smith, ‘Medical Audit’, *BMJ*, 1990, 300, 65.

83 Walshe, ‘Medical Accidents in the UK’; for an exception, see J. Bennett and K. Walshe, ‘Occurrence Screening as a Method of Audit’, *BMJ*, 1990, 300, 1248–51.

84 According to the Chief Executive of the King Edward’s Hospital Fund, Robert Maxwell, in 1984, the British medical profession appeared ‘collectively allergic’ to medical audit. R.J. Maxwell, ‘Quality Assessment in Health’, *British Medical Journal (Clinical Research Ed.)*, 1984, 288, 1470–72. For further discussion of audit, see Moore, *Managing Diabetes*.

85 K. Gannon, ‘Medical Accidents: Attitudes and Opinions of Doctors’, *International Journal of Risk & Safety in Medicine*, 1992, 3, 253–62.

86 Vincent, ‘Risk, Safety, and the Dark Side of Quality’.

87 Vincent et al., ‘Medical Accidents’; Charles Vincent, Maeve Ennis, and Robert J. Audley, *Medical Accidents* (Oxford: Oxford University Press, 1993); G. Neale, ‘Clinical Analysis of 100 Medicolegal Cases’, *BMJ*, 1993, 307, 1483–87; Vincent, Neale, and Woloshynowych, ‘Adverse Events in British Hospitals’.

88 Mold, *Making the Patient-Consumer*.

89 Mulcahy, *Disputing Doctors*.

90 NHS Reorganisation Act 1973, schedule 3.

91 Klein, *The New Politics of the NHS*, 61.

92
*Hatcher v Black*, *The Times*, 2 July 1954, quoted in Ewoud Hondius, *The Development of Medical Liability* (Cambridge: Cambridge University Press, 2014).

93 Edward Pilkington, ‘When Doctors Go on the Defensive’, *The Guardian*, 25 April 1990.

94 TNA MH 99/154, ‘National Health Service. Legal Proceedings. H.M.54 (32)’, 1954.

95 R. Dingwall and P. Fenn, ‘Is NHS Indemnity Working and Is There a Better Way?’, *British Journal of Anaesthesia*, 1994, 73, 69–77.

96 Simanowitz, *The Man Under the Clapham Omnibus*.

97 Interview with Arnold Simanowitz, Chief Executive, AVMA (1982–2003), 29 September 2020. In 2003, AVMA was renamed Action against Medical Accidents (AvMA).

98 Ibid.

99 Hansard, HC Deb 1 February 1991, vol. 174 col. 1271.

100 Simanowitz, *The Man Under the Clapham Omnibus*.

101 Maeve Ennis and Charles Vincent, ‘The Effects of Medical Accidents and Litigation on Doctors and Patients’, *Law & Policy*, 1994, 16, 97–121.

102 Dingwall and Fenn, ‘Is NHS Indemnity Working’.

103 Quoted in Alleen Ballantyne, ‘Doctors Reject Legal Liability for NHS Cuts: Conditions Are “Becoming Dangerous to Patient Care”’, *The Guardian*, 9 August 1988.

104 For example, Anon., ‘The Trio of Tragedy’, *The Guardian*, 3 August 1988.

105 Modern Records Centre, University of Warwick, MSS.184/1/13/13, ‘Medical accidents: Issued by the Thames Television “Help!” Programme’, 1983.

106 For example, Andrew Veitch, ‘Student whose life was ruined by doctors’ blunder wins 1 million pounds’, *The Guardian*, 11 July 1987.

107 Dingwall and Fenn, ‘Is NHS Indemnity Working’.

108 National Health Service (Compensation) Bill, 1991.

109 Dingwall and Fenn, ‘Is NHS Indemnity Working’.

110 NHS Executive, *Risk Management in the NHS* (London: Department of Health, 1993).

111 Charles Vincent, *Clinical Risk Management* (London: BMJ Publishing Group, 1995); Charles Vincent and Roger V. Clements, ‘Clinical Risk Management—Why Do We Need It?’, *AVMA Medical & Legal Journal*, 1995, 1, 1–4.

112 Vincent, Ennis, and Audley, *Medical Accidents*.

113 J. Mant and A. Gatherer, ‘Managing Clinical Risk’, *BMJ*, 1994, 308, 1522–23.

114 Paul Fenn and Tom Egan, ‘Risk Management in the NHS: Governance, Finance and Clinical Risk’, *Clinical Medicine*, 2012, 12, 25–28.

115 Martin, *Hospitals in Trouble*; Louise Hide and Joanna Bourke, ‘Cultures of Harm in Institutions of Care: Introduction’, *Social History of Medicine*, 2018, 31, 679–87; Claire Hilton, ‘A Tale of Two Inquiries: *Sans Everything* and Ely’, *The Political Quarterly*, 2019, 90, 185–93.

116 Julia Hartley-Brewer, ‘Downfall of Surgeon Who Ruined Lives’, *The Guardian*, 2 June 2000.

117 Dixon-Woods, Yeung, and Bosk, ‘Why Is UK Medicine’.

118 See Fay Bound Alberti, *Matters of the Heart: History, Medicine, and Emotion* (Oxford: Oxford University Press, 2010).

119 Ian Murray, ‘Parents Want Inquiry over Surgery Deaths’, *The Times*, 19 February 1998; Jeremy Laurance, ‘Parents of Heart Children Call for New Inquiry’, *The Independent*, 19 February 1998.

120 Walter Ellis, ‘Focus: Doctors in the Dock’, *The Independent*, 18 June 2000.

121 Richard Smith, ‘All Changed, Changed Utterly: British Medicine Will Be Transformed by the Bristol Case’, *BMJ*, 1998, 316, 1917–18.

122 Dixon-Woods, Yeung, and Bosk, ‘Why is UK Medicine’.

123 DOH, *The New NHS: Modern, Dependable*, Cm. 2807 (London: TSO, 1997).

124 DOH, *A First Class Service: Quality in the New NHS* (London: Department of Health, 1998), 3.2; Berwick, ‘Continuous Improvement’.

125 DOH, *First Class Service*.

126 DOH, *Organisation with a Memory*.

127 Wears and Sutcliffe, *Still Not Safe*. The concept of ‘safety culture’ was first introduced by the International Nuclear Safety Advisory Group (INSAG) following the Chernobyl Disaster in 1986. International Nuclear Safety Advisory Group, *Summary Report on the Post-Accident Review Meeting on the Chernobyl Accident*. Safety Series No. 75-INSAG-1 (Vienna: International Atomic Energy Agency, 1986).

128 Christopher Sirrs, ‘Health and Safety in the British Regulatory State, 1961–2001: The HSC, HSE and the Management of Occupational Risk’ (unpublished PhD thesis, London School of Hygiene and Tropical Medicine, 2016).

129 Reason, *Human Error*; Reason, *Managing the Risks of Organizational Accidents*.

130 Wears and Sutcliffe, *Still Not Safe*.

131 TNA JA 440/88, ‘Expert Group on Learning from Experience in the NHS: Note of First Meeting, 8 February 1999, Richmond House’, 1999.

132 House of Commons Health Committee, *Sixth Report. Procedures Related to Adverse Clinical Incidents and Outcomes in Medical Care. Volume 1. Report and Proceedings of the Committee*, HC 549-I, 1999.

133 The absence of this duty was later confirmed by the Court of Appeal in 1997, and the European Court of Human Rights, who determined: ‘As the law stands now … doctors have no duty to give the parents of a child who died as a result of their negligence a truthful account of the circumstances of the death, nor even to refrain from deliberately falsifying records.’ (European Court of Human Rights, Third Section Decision, William and Anita Powell against the United Kingdom, 4 May 2000). To this date there remains no statutory duty of candour covering all healthcare professionals. Interview with Will Powell, 16 July 2021.

134 TNA JA 440/90, E.H. Ryan, ‘Health Select Committee: Report into Procedures Related to Adverse Clinical Incidents and Outcomes in Medical Care’, 11 November 1999; House of Commons Health Committee, *Sixth Report*.

135 TNA JA 440/87/1, E.H. Ryan, ‘Health Select Committee Inquiry: Adverse Incident Procedures’, 7 June 1999.

136 DOH, *Organisation with a Memory*; DOH, *Building a Safer NHS for Patients: Implementing ‘An Organisation with a Memory’* (London: Department of Health, 2001).

137 Stacey, ‘The General Medical Council and Professional Self-Regulation’.

138 Christopher Hood, ‘A Public Management for all Seasons?’, *Public Administration*, 1991, 69, 3–19; Power, *The Audit Society*.

139 Harrison and Wood, ‘Scientific-Bureaucratic Medicine’.

140 Kohn et al, *To Err is Human*.

141 Jeremy Laurance, ‘Surgeons Should Train on Simulators, like Pilots’, *The Independent*, 16 March 2009.

142 Quick, *Regulating Patient Safety*.

143 Robert Francis QC (chair), *Report of the Mid Staffordshire NHS Foundation Trust Public Inquiry. Volume 1: Analysis of Evidence and Lessons Learned (Part 1*), HC 898-1 (London: Stationery Office, 2013), 10.

144 See, for instance, Robert Francis QC, *Freedom to Speak Up: An Independent Review into Creating an Open and Honest Reporting Culture in the NHS*, February 2015.

## Notes

At the time of writing, Dr Christopher Sirrs was a Research Fellow at the Department of History, University of Warwick. His postdoctoral research project, ‘Hazardous Hospitals’ (2020–23), explored the history of safety in NHS general hospitals. Sirrs’ prior research included the history of occupational health and safety regulation in Britain, and the intellectual and policy history of health systems.

